# Epidemiology of Acute Chagas Disease in the Amazon: Association between the açaí production chain and case reports in Pará state, Brazil

**DOI:** 10.1590/0037-8682-0360-2025

**Published:** 2026-03-30

**Authors:** Mateus Gomes Oliveira, André Walsh-Monteiro, John Fontenele Araújo, Marco Aurélio Oliveira

**Affiliations:** 1 Instituto Federal do Pará, Laboratório de Neuroquímica e Comportamento, Tucuruí, PA, Brasil.; 2 Universidade Federal do Rio Grande do Norte, Departamento de Fisiologia, Laboratório de Neurobiologia e Ritmicidade Biológica, Natal, RN, Brasil.; 3 Universidade Federal do Delta do Parnaíba, Parnaíba, PI, Brasil.; 4 Instituto Federal do Tocantins, Coordenação de Educação Física, Laboratório de Psicobiologia e Exercício Físico, Araguatins, TO, Brasil.; 5 Universidade Federal do Rio Grande do Norte, Programa de Pós-Graduação em Psicobiologia, Natal, RN, Brasil.

**Keywords:** Chagas Disease, Epidemiology, Oral transmission, Açaí, Seasonality, Pará

## Abstract

**Background:**

Acute Chagas Disease (ACD) in the Brazilian Amazon exhibits a unique epidemiological profile characterized primarily by oral transmission. The state of Pará, recognized as the world's largest producer of açaí, represents an epicenter of the disease. This study aimed to analyze the epidemiological patterns of ACD cases in Pará and investigate the relationship between its seasonality and the açaí production chain.

**Methods:**

This epidemiological study used data on 2,762 ACD cases reported to the Notifiable Diseases Information System (SINAN) between 2013 and 2023. Sociodemographic and epidemiological variables were evaluated. Time series analysis was performed to determine seasonality and assess its correlation with variations in açaí prices.

**Results:**

Oral transmission accounted for 87.44% of cases. The predominant demographic profiles were males (54.16%) and individuals with parda (mixed-race) skin color (85.41%). Temporal analysis revealed a significant seasonal trend (p < 0.001), with a peak incidence projected at the end of September, coinciding with the açaí harvest season. A moderate negative Spearman correlation was observed between ACD case numbers and açaí prices (r = -0.413; p < 0.001), suggesting that increased supply and consumption are associated with a heightened risk of infection.

**Conclusions:**

The epidemiology of ACD in Pará is closely linked to the açaí production cycle, confirming its role as the principal mode of oral transmission in the region. These results underscore the importance of public health policies focused on food safety and effective management practices throughout the entire açaí production chain to prevent new cases.

## INTRODUCTION

Chagas disease, caused by the flagellate protozoan *Trypanosoma cruzi*, is an anthropozoonotic vector-borne infection recognized as one of the most neglected tropical diseases in Latin America^1^. Although the classic mode of transmission is vectorial-through contact with the feces of infected triatomine bugs (commonly known as "kissing bugs")-other significant epidemiological routes include oral transmission, organ transplantation, blood transfusion, congenital infection, and laboratory accidents^2^. The disease progresses through two phases: an acute phase (ACD), which is frequently asymptomatic or presents with nonspecific symptoms; and a chronic phase, which may progress to severe cardiac and digestive complications, representing a considerable public health challenge^3^.

Although the presence of *Triatoma rubrofasciata* was initially reported in the state of Pará in 1910^4^, confirmation that the parasite (*T. cruzi*) circulated among sylvatic vectors ("kissing bugs") and wild animal reservoirs in the region only emerged in 1949^5^. This discovery demonstrated the existence of a natural transmission cycle, affirming the potential risk for human infection. The first documented evidence of this risk was provided by Jeffrey Shaw^6^, who reported autochthonous acute cases within a family residing in the Canudos neighborhood of Belém. Despite extensive triatomine testing across 409 residences-including five consecutive nights in the affected family's well-constructed masonry house-none were found. The cluster of acute cases within a single household suggested a high level of local infestation, though this was not substantiated by entomological surveys. The identification of these initial autochthonous ACD cases in Pará and other regions highlighted a new and emerging transmission pattern.

In Brazil, the distribution of Chagas disease is heterogeneous, with the Amazon Region now serving as the main endemic area for acute cases^7^. Transmission in this area is distinct, predominantly occurring via the oral route, often linked to the consumption of foods contaminated with triatomines or their excreta^8^. This form of transmission is particularly concerning in the Amazon, where outbreaks are frequently associated with the consumption of juices or pulps from native fruits, such as açaí (*Euterpe oleracea* and *Euterpe precatoria*) and bacaba (*Oenocarpus bacaba*), as well as contaminated game meat^9^ ^,^ ^10^. This context poses unique challenges for surveillance and control, necessitating prevention strategies that account for local cultural and ecological nuances. 

Within this framework, the state of Pará is the epicenter of ACD in Brazil, accounting for approximately 86% of reported cases and a substantial proportion of oral transmission outbreaks^11^. Existing literature underscores that this transmission route is intrinsically linked to food consumption, particularly açaí (*Euterpe oleracea*), a fruit of immense regional cultural and economic relevance. Contamination can occur during harvesting, transportation, or artisanal processing, presenting ongoing public health concerns and intersecting issues of extractivist practices, food safety, and disease dynamics^12^ ^,^ ^13^.

Despite strong evidence associating ACD with the açaí supply chain, further research is required to clarify the temporal patterns and magnitude of this relationship. Accordingly, this study aims to characterize the epidemiological profile of ACD in Pará from 2013 to 2023 and to evaluate the ecological correlation between its seasonal pattern and fluctuations in açaí prices.

## METHODS

### Data Sources

This descriptive, quantitative study used publicly available secondary data to analyze the epidemiological profile of ACD. Data on reported cases were sourced from SINAN (Notifiable Diseases Information System), accessible through the DATASUS (Department of Informatics of the Unified Health System) online portal^14^. 

The study included ACD cases reported in Brazil between January 2013 and December 2023, focusing specifically on residents of the state of Pará. The variables analyzed in SINAN included sociodemographic factors (sex, age group, and race/skin color), clinical features (probable mode and location of infection, confirmation criteria, outcome), and temporal aspects (month and year of notification). 

Historical açaí price data were obtained from the National Supply Company (CONAB) portal^15^. This series represented monthly nominal prices (BRL/kg) received by producers in Pará from January 2014 to December 2023. No inflation adjustments or moving averages were applied. The price data were temporally aligned with monthly ACD notifications for correlation and COSINOR analysis.

### Analyses

Excel was used to tabulate the data. CosinorOnline^16^, coded in PHP and JavaScript, was applied for rhythm analysis, while Spearman’s rank correlation test was conducted using Jamovi (version 2.5)^17^ to evaluate the association between ACD cases and açaí prices. Variables with missing values (“Ignored/Blank”) were included in descriptive summaries to accurately reflect data completeness; however, these entries were excluded from inferential statistical analyses and time-series evaluations to minimize potential bias. ACD incidence rates were calculated per 10,000 inhabitants from January 2013 to December 2023, and stratified by age group and municipality. Population denominators were drawn from the 2022 Demographic Census, provided by the Brazilian Institute of Geography and Statistics^18^. 

The COSINOR (COSIne + NORmal) method was employed to assess the circannual rhythmicity of ACD notifications^19^. This method is designed to analyze time-series data to detect and quantify biological rhythms and other cyclical phenomena by fitting a cosine curve (sine wave) to the observed data over time. The mathematical model estimates three primary parameters: MESOR (Midline Estimating Statistic of Rhythm), which represents the mean value around which the data oscillate, analogous to an arithmetic mean but adjusted for rhythmicity; Amplitude (A), quantifying the wave’s magnitude ("height") as half the difference between the peak and trough values; and Acrophase (φ), denoting the timing of cosine curve’s peak, indicating the period of the greatest activity, typically expressed in hours or degrees (with 360° corresponding to a full cycle, such as 24 h). 

Statistical significance was assessed using an F-test and the amplitude test to compute the probability (p-value). Cosine curves with a fixed 12-month period were fitted to the monthly time series for both ACD notifications and açaí prices, enabling identification and estimation of key parameters: mesor (mean level), amplitude (variation around the mesor), acrophase (peak occurrence), and bathyphase (lowest incidence phase). A time series was deemed significant if its amplitude differed from zero. Application of the COSINOR method enabled evaluation of consistent annual rhythmicity in disease occurrence.

For all analyses, age groups adhered to the aggregations defined by SINAN. Missing data-primarily recorded as "Ignored/Blank" for variables, such as race/skin color and probable location of infection-were retained for analyses to accurately represent the integrity of SINAN records. The significance threshold adopted for statistical tests was set at p < 0.05.

## RESULTS

Data analysis facilitated a comprehensive description of the demographic, clinical, and epidemiological profiles of patients affected by ACD. Between 2013 and 2023, 2,762 cases of ACD were registered in the state of Pará. Among these, 1,496 (54.16%) occurred in males and 1,266 (45.84%) in females. The most prevalent age group was 20-39 years, accounting for 957 cases (34.65%), followed by the 40-59 age group with 666 cases (24.15%; Table 1). The lowest frequencies were found in the under 1 year (n = 23, 0.83%) and over 80 years (n = 25, 0.91%) groups. Regarding race/skin color, individuals identified as Parda (mixed race) predominated, representing 2,359 cases (85.41%), followed by White with 196 cases (7.10%). Other groups included Ignored/Blank (n = 49, 1.77%), Black (n = 135, 4.89%), Asian (n = 10, 0.36%), and Indigenous (n = 13, 0.47%).

**TABLE 1:** Descriptive data. Acute Chagas disease in the state of Pará between 2013 and 2023

		n	%
Total		2,762	100
**Sex**	Male	1,496	54.16
	Female	1,266	45.84
**Age group (years)**	< 1	**23**	**0.83**
	1-4	125	4.53
	5-9	214	7.75
	10-14	240	8.70
	15-19	256	9.27
	20-39	957	34.65
	40-59	666	24.15
	60-64	96	3.48
	65-69	74	2.68
	70-79	86	3.11
	> 80	25	0.91
**Race/skin color**	Ignored/Blank	49	1.77
	White	196	7.10
	Black	135	4.89
	Asian	10	0.36
	Parda (Mixed-race)	2,359	85.41
	Indigenous	13	0.47
**Probable mode of infection**	Ignored/Blank	182	6.59
	Vector-borne	159	5.76
	Vertical	1	0.04
	Accidental	4	0.14
	Oral	2,415	87.44
	Other	1	0.04
**Probable location of infection**	Ignored/Blank	744	26.94
	Blood bank	10	0.36
	Household	1,873	67.81
	Laboratory	1	0.04
	Other	134	4.85
**Confirmation criteria**	Ignored/Blank	33	1.19
	Laboratory	2,646	95.80
	Clinical-epidemiological	80	2.90
	Under investigation	3	0.11
**Outcome**	Ignored/Blank	281	10.17
	Alive	2,437	88.23
	Death by reported disease	34	1.23
	Death by other cause	10	0.36

The primary probable mode of infection was oral transmission, (2,415 cases; 87.44%), whereas vector-borne transmission accounted for 159 cases (5.76%). Additional modes included Ignored/Blank (n = 182, 6.59%), vertical (n = 1, 0.04%), accidental (n = 4, 0.14%), and other (n = 1, 0.04%). Most infections were presumed to occur within households (1,873 cases; 67.81%), followed by Ignored/Blank responses (n = 744, 26.94%). Other probable sites included blood banks (n = 10, 0.36%), laboratories (n = 1, 0.04%), and other locations (n = 134, 4.85%). 

Laboratory confirmation was obtained in 2,646 cases (n = 2.646, 95.80%), whereas 80 cases (2.90%) were confirmed through clinical epidemiological criteria. An additional 33 cases (1.19%) were categorized as Ignored/Blank, and 3 (0.11%) remained under investigation. Of the 2,762 cases, 2,437 (88.23%) were reported as alive, 34 (1.23%) died from the reported disease, 10 (0.36%) died from other causes, and outcomes were undetermined or not reported in 281 patients (10.17%).

**FIGURE 1:**
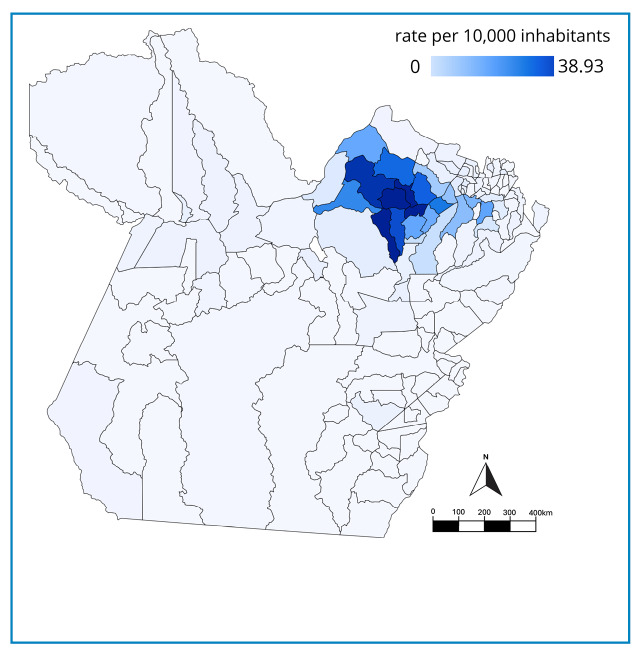
Acute Chagas Disease rate per 10,000 inhabitants by municipality in the state of Pará, from 2013 to 2023.

**FIGURE 2:**
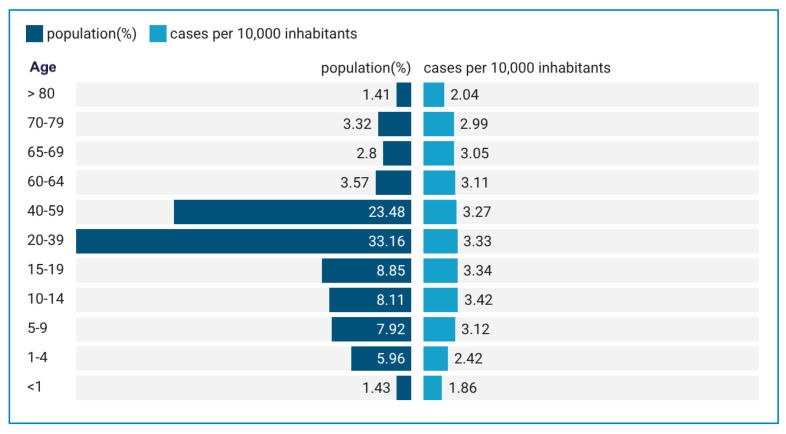
Rate of reported Acute Chagas Disease cases per 10,000 inhabitants by age group in the state of Pará from 2013 to 2023.

The lowest incidence rate was observed in the < 1-year age group (1.86 per 10,000 inhabitants). Across municipalities, the ACD incidence rate per 10,000 inhabitants in Pará exhibited notable variability. The overall incidence rate for the period studied was 3.40 per 10,000 inhabitants, with rates of 3.69 and 3.11 per 10,000 inhabitants for males and females, respectively. High-incidence municipalities, such as Limoeiro do Ajuru (37.20 per 10,000 inhabitants) and Curralinho (38.93 per 10,000 inhabitants), are located in the Baixo Tocantins mesoregion (Figure 1). Incidences across age groups indicated the highest values in the 10-14 (3.42 per 10,000 inhabitants), 15-19 (3.34 per 10,000) and 20-39 (3.33 per 10, 000) age groups (Figure 2). 

**FIGURE 3:**
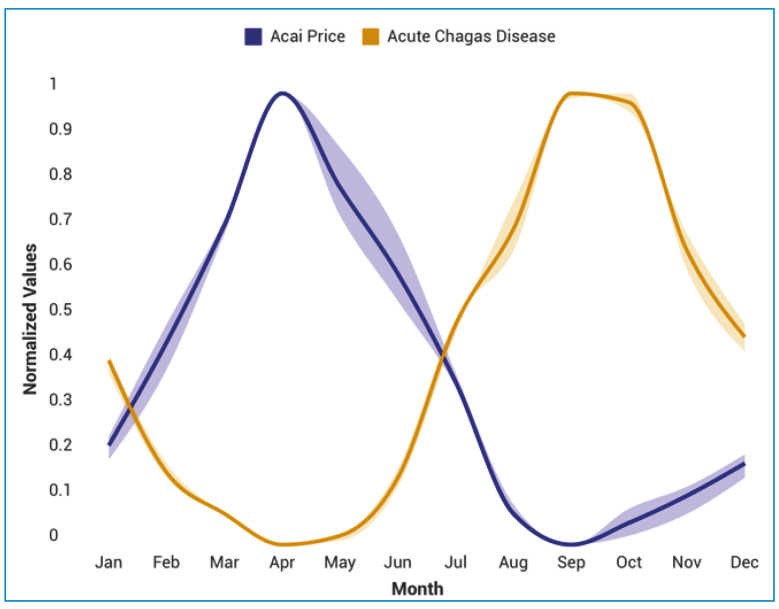
Time series of reported Acute Chagas Disease (ACD) cases and açaí sale price (BRL/kg) in the state of Pará from Jan/2014 to Dec/2023. Data are normalized and presented with standard error. COSINOR analysis shows ACD notifications with an acrophase at 9.73, at the end of September and a bathyphase at 3.73 (F = 46.01; p = 0.00001), at the end of March; for the açaí price, the acrophase is at 4.50 and the bathyphase at 10.50 (F = 28.87; p = 0.00001), consistent with an antiphase rhythmic pattern between the two variables. The variables show a negative correlation (r = -0.413; p < 0.001) according to the Spearman test.

**FIGURE 4:**
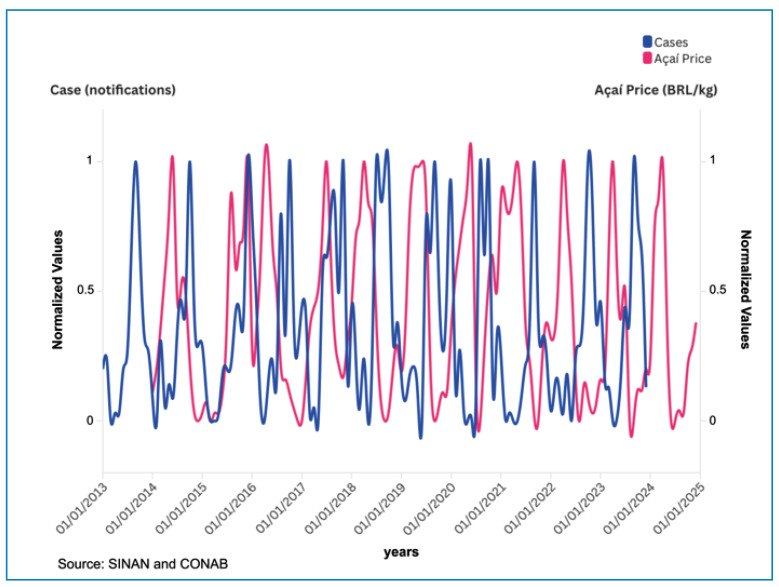
Time series of reported Acute Chagas Disease (ACD) cases (2013-2023) and the price received by the producer in the state of Pará (2014-2023). Data originate from SINAN and CONAB.

A noticeable seasonal pattern was evident in the temporal distribution of cases in Pará, with a higher concentration of cases during the açaí harvest season, which aligns with periods when the fruit market’s price declines in the region (Figures 3 and 4).

## DISCUSSION

This study characterized the epidemiological profile of 2,762 cases of ACD in the State of Pará from 2013 to 2023, reaffirming the disease’s prominence in Brazil. The primary finding was the high proportion of probable oral transmission (87.44%), consistent with the Amazonian transmission pattern characterized by strong associations with environmental and behavioral factors. These results underscore the need for public health interventions tailored to this unique epidemiological context.

The sociodemographic analysis revealed that affected individuals were predominantly male (54.16%) and self-declared as parda (mixed race; 85.41%), reflecting the state's demographic composition^20^. While the largest absolute number of cases occurred among the economically active population (20-39 years), the highest incidence rate was observed in adolescents aged 10-14 years (3.42 per 10,000 inhabitants). These findings suggest that early and widespread exposure may be associated with a common source of infection at the community and family levels, aligning with patterns of foodborne transmission.

The predominance of oral transmission is likely attributable to exposure to contaminated foods consumed within households and communities. Indeed, a significant proportion of infections (67.81%) occurred in domestic settings, largely due to the consumption of contaminated foods, such as açaí and bacaba^21^. This transmission route aligns with the epidemiology of ACD in Pará and local dietary and cultural practices, particularly those involving the preparation and consumption of raw plant-based products like açaí, which are integral to the Amazonian diet. Consequently, outbreaks linked to oral transmission have increased, reflecting sanitation challenges associated with food handling and preparation-often performed artisanally and consumed within family and community groups in rural and riverine areas^22^. The reliance on a food vector also introduces temporal regularity, with case occurrences closely linked to harvest cycles.

This temporal pattern was validated by time-series analysis, which revealed a significant seasonal rhythm in ACD incidence (p < 0.001), with the acrophase estimated at the end of September. This coincides with the latter half of the year, corresponding to the açaí (*E. oleracea*) harvest season in Pará, spanning June to December^23^. Furthermore, a statistically significant negative correlation between case numbers and the average price of açaí further reinforces this association, considering an incubation period of 3-22 days and mandatory reporting within 24 h to municipal and state health secretariats via SINAN^24^. Reduced prices during the harvest season increases accessibility, likely increasing consumption and subsequently raising the risk of exposure to *Trypanosoma cruzi* through vector-borne (5.76%) and oral (87.44%) routes, particularly when fruits are improperly prepared and do not adhere to sanitary standards^25^. This seasonal-economic connection is fundamental for designing effective surveillance strategies, which should be intensified during the months preceding and coinciding with peak transmission.

Parallel to the temporal distribution, spatial analysis demonstrated notable heterogeneity in disease occurrence across the state. While reports of cases have been made in multiple regions, ACD is concentrated in high-endemicity zones, primarily in cities where the fruit is cultivated and extracted. Our findings support this: of the fourteen cities identified by the Amazon Foundation for the Support of Studies and Research of Pará (FAPESPA) as açaí production specialists^26^, ten-São Sebastião da Boa Vista, Curralinho, Limoeiro do Ajuru, Bagre, Breves, Oeiras do Pará, Anajás, Abaetetuba, Cametá, and Igarapé-Miri-rank among those with the highest ACD rates per 10,000 inhabitants, with rates ranging from 39 to 14.5 (Supplementary Material files). These values significantly exceed the state (3.4 per 10,000 inhabitants) and national (0.176 per 10,000 inhabitants) averages. The affected cities are located in the Furo de Breves, Portel, and Cametá microregions. Mapping these highly vulnerable areas is thus an essential strategy to enhance resource allocation and target both control measures and health surveillance where they are most critical. Municipal heterogeneity likely reflects differences in socioeconomic conditions, dependence on extractive activities, riverine settlement patterns, and access to sanitation and health services. High-incidence municipalities are predominantly located in floodplain and riverine environments, where açaí extraction and informal commercialization are central to local economies, potentially increasing exposure to contaminated products.

Regarding clinical outcomes, the data indicated a predominantly positive outcome, with 88.23% of patients alive at the time of notification, suggesting that the state's health system effectively diagnoses and treats the acute phase of ACD. Although Pará reports the highest number of acute cases, it does not have the highest standardized rates of hospitalization for ICD-10 B57, mortality from cardiac causes, or heart failure hospitalizations^27^. The state boasts the nation’s highest estimated population coverage by primary care teams and the highest rate of Chagas disease-related outpatient procedures per capita^27^. However, the 1.23% case fatality rate among those with recorded outcomes is a significant indicator that should not be overlooked. Although these percentages are low, they carry meaningful adverse public health consequences and highlight that, despite an overall positive impact, ACD poses a considerable risk of severe complications and permanent sequelae. This supports the continued prioritization of ACD surveillance and control.

These findings underscore the importance of state decree 326/2012^25^, which governs good manufacturing practices for açaí processing in Pará. However, ensuring effective implementation remains challenging, particularly in remote riverine regions where artisanal processing predominates, and inspection resources are constrained. Strengthening integrated actions among sanitary surveillance, epidemiological surveillance, and the agricultural sector-particularly during peak harvest seasons-may support earlier outbreak detection and mitigate foodborne transmission risks. Future research should adopt production-chain traceability methods and conduct direct microbiological and entomological investigations at harvesting, processing, and commercialization sites to better characterize contamination routes and identify essential intervention control points.

It is essential to recognize the limitations inherent in this study. First, the completeness of secondary data sourced from SINAN was variable, with a proportion of records containing missing or “Ignored/Blank” entries, particularly for variables such as probable location of infection and outcome. To minimize potential bias, these categories were retained only for descriptive analyses to transparently illustrate data quality, but were excluded from all inferential and time-series analyses, including correlation and COSINOR modeling. Nevertheless, incomplete reporting may impact the accuracy and generalizability of certain estimates, which should be considered when interpreting the results. 

A key consideration of this research is that the association between ACD and açaí consumption was established through ecological analysis using correlations with açaí prices. Notably, the observed correlation reflects an ecological association rather than a direct causal relationship. While numerous outbreaks of oral transmission have been linked to Chagas disease^22^, the price of açaí functions as an indirect measure of production, availability, and consumption patterns, and cannot directly assess individual exposure or contamination risk due to the multifactorial nature of contamination in the Amazon. Additionally, although vector-borne transmission accounts for 5.76% of reported cases, there is no additional information regarding the specific location of transmission, such as whether it occurred at home or in a wooded area. The months with the highest incidence rates also coincide with the hottest periods of the year, which contribute to regional wildfires and increased vector activity^28^.

An important avenue for future research on the relationship between ACD and açaí consumption is to distinguish between the two fruit species: *E. oleracea,* found in the eastern Amazon within the states of Pará, Amapá, Tocantins, and Maranhão, and *Euterpe precatoria*, primarily distributed across Pará, Amazonas, Acre, and Rondônia^29^. These species exhibit distinct harvest periods; *E. oleracea* yields a greater proportion of mature bunches (56%) at the end of the dry season from October to December, whereas *Euterpe precatoria* shows increased production (49%) at the conclusion of the rainy season from May and July^30^. Notably, most açaí produced in Pará derives from *E. oleracea*, which dominates native floodplains and igapós, key extraction zones, as well cultivated upland varieties developed by Empresa Brasileira de Pesquisa Agropecuária (EMBRAPA), such as BRS Pará and BRS Pai d'Égua, both improved varieties of this species^29^. Therefore, while current evidence strongly supports this association, it should be viewed as a robust indicator warranting further investigation through specifically designed studies to confirm the relationship at the individual level.

## CONCLUSION

In conclusion, this study demonstrates that the epidemiology of ACD in Pará is inextricably associated with, and significantly influenced by, the açaí production chain. The predominance of oral transmission, spatial clustering of cases within producing areas, and marked seasonality corresponding with the harvest season confirm that fruit serves as the primary vehicle for *T. cruzi* transmission in this region. These findings reinforce the urgent need for public health policies that integrate epidemiological surveillance with food safety, including producer training, sanitary inspections, and health education initiatives. Accordingly, intersectoral actions are imperative to mitigate risk and control the disease in this unique context.
