# Supplementary material for: Epidemiology of Acute Chagas Disease in the Amazon: Association between the açaí production chain and case reports in Pará state, Brazil
Source: Rev Soc Bras Med Trop. 2026 Mar 30;59:e0360-2025. doi: 10.1590/0037-8682-0360-2025 (PMC13035243; doi:10.1590/0037-8682-0360-2025)
Supplement: Supplementary material [file 1678-9849-rsbmt-59-e0360-2025-md1.pdf]

SUPPLEMENTARY FILE

Rate of Acute Chagas Disease by municipality in the state of Pará between 2013 and 2023 per 10,000 inhabitants

	Sex	Inhabitants	Cases	Tax
**Country**				
	Total	203,080,756	3571	0.176
Brazil	Male	98,532,431	1931	0.196
	Female	104,548,325	1640	0.157
**State**				
	Total	8,120,131	2762	3.40
Pará	Male	4,051,813	1496	3.69
	Female	4,068,318	1266	3.11
**City**				
	Total	158,188	353	22.32
Abaetetuba (PA)	Male	79,297	175	22.07

	Female	78,891	178	22.56
	Total	6,136	0	0.00
Abel Figueiredo (PA)	Male	3,113	0	0.00
	Female	3,023	0	0.00
	Total	59,023	61	10.33
Acará (PA)	Male	30,551	35	0.00
	Female	28,472	26	9.13
	Total	37,765	57	15.09
Afuá (PA)	Male	19,708	35	17.76
	Female	18,057	22	12.18
	Total	18,080	2	1.11
Água Azul do Norte (PA)	Male	9,696	2	2.06
	Female	8,384	0	0.00
	Total	69,377	3	0.43
Alenquer (PA)	Male	35,689	3	0.84

	Female	33,688	0	0.00
	Total	34,280	0	0.00
Almeirim (PA)	Male	17,647	0	0.00
	Female	16,633	0	0.00
	Total	126,279	3	0.24
Altamira (PA)	Male	62,942	2	0.32
	Female	63,337	1	0.16
	Total	28,011	67	23.92
Anajás (PA)	Male	14,508	35	24.12
	Female	13,503	32	23.70
	Total	478,778	64	1.34
Ananindeua (PA)	Male	227,304	26	1.14
	Female	251,474	38	1.51
Anapu (PA)	Total	31,850	0	0.00
	Male	16,605	0	0.00

	Female	15,245	0	0.00
	Total	44,573	4	0.90
Augusto Corrêa (PA)	Male	22,797	4	1.75
	Female	21,776	0	0.00
	Total	23,774	8	3.37
Aurora do Pará (PA)	Male	12,079	3	2.48
	Female	11,695	5	4.28
	Total	18,290	0	0.00
Aveiro (PA)	Male	9,586	0	0.00
	Female	8,704	0	0.00
	Total	31,892	109	34.18
Bagre (PA)	Male	16,253	59	36.30
	Female	15,639	50	31.97
Baião (PA)	Total	51,641	7	1.36
	Male	25,917	4	1.54

	Female	25,724	3	1.17
	Total	4,031	0	0.00
Bannach (PA)	Male	2,164	0	0.00
	Female	1,867	0	0.00
	Total	126,650	135	10.66
Barcarena (PA)	Male	63,394	65	10.25
	Female	63,256	70	11.07
	Total	1,303,403	282	2.16
Belém (PA)	Male	610,777	131	2.14
	Female	692,626	151	2.18
	Total	18,099	0	0.00
Belterra (PA)	Male	9,164	0	0.00
	Female	8,935	0	0.00
Benevides (PA)	Total	63,567	10	1.57
	Male	31,251	5	1.60

	Female	32,316	5	1.55
Bom Jesus do Tocantins (PA)	Total	18,005	0	0.00
	Male	9,969	0	0.00
	Female	8,036	0	0.00
Bonito (PA)	Total	12,622	0	0.00
	Male	6,477	0	0.00
	Female	6,145	0	0.00
Bragança (PA)	Total	123,082	17	1.38
	Male	61,581	12	1.95
	Female	61,501	5	0.81
Brasil Novo (PA)	Total	24,718	0	0.00
	Male	12,635	0	0.00
	Female	12,083	0	0.00
Brejo Grande do Araguaia (PA)	Total	6,783	0	0.00
	Male	3,439	0	0.00

	Female	3,344	0	0.00
Breu Branco (PA)	Total	45,712	1	0.22
	Male	23,100	0	0.00
	Female	22,612	1	0.44
Breves (PA)	Total	106,968	321	30.01
	Male	55,278	186	33.65
	Female	51,690	135	26.12
Bujaru (PA)	Total	24,383	32	13.12
	Male	12,353	16	12.95
	Female	12,030	16	13.30
Cachoeira do Piriá (PA)	Total	19,630	1	0.51
	Male	10,351	0	0.00
	Female	9,279	1	1.08
Cachoeira do Arari (PA)	Total	23,981	3	1.25
	Male	12,126	1	0.82

	Female	11,855	2	1.69
	Total	134,184	207	15.43
Cametá (PA)	Male	68,840	120	17.43
	Female	65,344	87	13.31
	Total	77,079	1	0.13
Canaã dos Carajás (PA)	Male	39,387	0	0.00
	Female	37,692	1	0.27
	Total	70,394	2	0.28
Capanema (PA)	Male	34,350	1	0.29
	Female	36,044	1	0.28
	Total	56,506	2	0.35
Capitão Poço (PA)	Male	28,671	1	0.35
	Female	27,835	1	0.36
Castanhal (PA)	Total	192,256	15	0.78
	Male	92,506	10	1.08

	Female	99,750	5	0.50
	Total	20,757	1	0.48
Chaves (PA)	Male	11,118	1	0.90
	Female	9,639	0	0.00
	Total	12,868	2	1.55
Colares (PA)	Male	6,446	0	0.00
	Female	6,422	2	3.11
	Total	44,617	0	0.00
Conceição do Araguaia (PA)	Male	22,490	0	0.00
	Female	22,127	0	0.00
	Total	26,881	1	0.37
Concórdia do Pará (PA)	Male	13,550	0	0.00
	Female	13,331	1	0.75
	Total	14,036	0	0.00
Cumaru do Norte (PA)	Male	7,706	0	0.00

	Female	6,330	0	0.00
	Total	19,950	0	0.00
Curionópolis (PA)	Male	10,196	0	0.00
	Female	9,754	0	0.00
	Total	33,903	132	38.93
Curralinho (PA)	Male	17,863	69	38.63
	Female	16,040	63	39.28
	Total	14,117	2	1.42
Curuá (PA)	Male	7,338	1	1.36
	Female	6,779	1	1.48
	Total	41,262	3	0.73
Curuçá (PA)	Male	20,745	3	1.45
	Female	20,517	0	0.00
Dom Eliseu (PA)	Total	58,484	0	0.00
	Male	28,907	0	0.00

	Female	29,577	0	0.00
	Total	28,192	0	0.00
Eldorado do Carajás (PA)	Male	14,443	0	0.00
	Female	13,749	0	0.00
	Total	8,728	0	0.00
Faro (PA)	Male	4,483	0	0.00
	Female	4,245	0	0.00
	Total	17,898	0	0.00
Floresta do Araguaia (PA)	Male	9,498	0	0.00
	Female	8,400	0	0.00
	Total	24,703	2	0.81
Garrafão do Norte (PA)	Male	12,694	2	1.58
	Female	12,009	0	0.00
	Total	26,362	0	0.00
Goianésia do Pará (PA)	Male	13,525	0	0.00

	Female	12,837	0	0.00
	Total	31,786	9	2.83
Gurupá (PA)	Male	16,707	7	4.19
	Female	15,079	2	1.33
	Total	35,797	1	0.28
Igarapé-Açu (PA)	Male	17,767	0	0.00
	Female	18,030	1	0.55
	Total	64,831	94	14.50
Igarapé-Miri (PA)	Male	33,274	55	16.53
	Female	31,557	39	12.36
	Total	10,325	1	0.97
Inhangapi (PA)	Male	5,229	0	0.00
	Female	5,096	1	1.96
	Total	30,329	3	0.99
Ipixuna do Pará (PA)	Male	15,637	3	1.92

	Female	14,692	0	0.00
	Total	30,955	7	2.26
Irituia (PA)	Male	15,911	2	1.26
	Female	15,044	5	3.32
	Total	123,314	0	0.00
Itaituba (PA)	Male	62,288	0	0.00
	Female	61,026	0	0.00
	Total	49,754	0	0.00
Itupiranga (PA)	Male	26,048	0	0.00
	Female	23,706	0	0.00
	Total	24,042	1	0.42
Jacareacanga (PA)	Male	12,560	1	0.80
	Female	11,482	0	0.00
Jacundá (PA)	Total	37,707	0	0.00
	Male	18,504	0	0.00

	Female	19,203	0	0.00
Juruti (PA)	Total	50,881	4	0.79
	Male	26,154	4	1.53
	Female	24,727	0	0.00
Limoeiro do Ajuru (PA)	Total	29,569	110	37.20
	Male	15,291	66	43.16
	Female	14,278	44	30.82
Mãe do Rio (PA)	Total	34,353	0	0.00
	Male	16,887	0	0.00
	Female	17,466	0	0.00
Magalhães Barata (PA)	Total	8,115	1	1.23
	Male	4,151	1	2.41
	Female	3,964	0	0.00
Marabá (PA)	Total	266,533	2	0.08
	Male	132,772	0	0.00

	Female	133,761	2	0.15
	Total	25,971	1	0.39
Maracanã (PA)	Male	13,292	1	0.75
	Female	12,679	0	0.00
	Total	26,573	0	0.00
Marapanim (PA)	Male	13,647	0	0.00
	Female	12,926	0	0.00
	Total	111,785	12	1.07
Marituba (PA)	Male	54,692	5	0.91
	Female	57,093	7	1.23
	Total	27,094	0	0.00
Medicilândia (PA)	Male	14,325	0	0.00
	Female	12,769	0	0.00
Melgaço (PA)	Total	27,881	56	20.09
	Male	14,568	30	20.59

	Female	13,313	6	4.51
	Total	27,198	13	4.78
Mocajuba (PA)	Male	13,800	4	2.90
	Female	13,398	9	6.72
	Total	84,094	41	4.88
Moju (PA)	Male	43,881	23	5.24
	Female	40,213	18	4.48
	Total	23,501	2	0.85
Mojuí dos Campos (PA)	Male	12,231	2	1.64
	Female	11,270	0	0.00
	Total	60,012	5	0.83
Monte Alegre (PA)	Male	30,351	3	0.99
	Female	29,661	2	0.67
Muaná (PA)	Total	45,368	113	24.91
	Male	22,997	64	27.83

	Female	22,371	49	21.90
	Total	20,478	0	0.00
Nova Esperança do Piriá (PA)	Male	10,516	0	0.00
	Female	9,962	0	0.00
	Total	13,955	0	0.00
Nova Ipixuna (PA)	Male	7,294	0	0.00
	Female	6,661	0	0.00
	Total	12,806	0	0.00
Nova Timboteua (PA)	Male	6,503	0	0.00
	Female	6,303	0	0.00
	Total	33,638	0	0.00
Novo Progresso (PA)	Male	17,487	0	0.00
	Female	16,151	0	0.00
Novo Repartimento (PA)	Total	60,732	4	0.66
	Male	31,309	2	0.64

	Female	29,423	2	0.68
	Total	52,229	0	0.00
Óbidos (PA)	Male	26,744	0	0.00
	Female	25,485	0	0.00
	Total	33,844	92	27.18
Oeiras do Pará (PA)	Male	17,643	51	28.91
	Female	16,201	41	25.31
	Total	68,294	1	0.15
Oriximiná (PA)	Male	34,456	1	0.29
	Female	33,838	0	0.00
	Total	17,855	1	0.56
Ourém (PA)	Male	9,047	0	0.00
	Female	8,808	1	1.14
Ourilândia do Norte (PA)	Total	32,467	0	0.00
	Male	16,542	0	0.00

	Female	15,925	0	0.00
	Total	41,097	1	0.24
Pacajá (PA)	Male	21,632	1	0.46
	Female	19,465	0	0.00
	Total	6,885	0	0.00
Palestina do Pará (PA)	Male	3,538	0	0.00
	Female	3,347	0	0.00
	Total	105,550	5	0.47
Paragominas (PA)	Male	52,461	2	0.38
	Female	53,089	3	0.57
	Total	267,836	1	0.04
Parauapebas (PA)	Male	133,922	0	0.00
	Female	133,914	1	0.07
Pau D'Arco (PA)	Total	6,931	0	0.00
	Male	3,555	0	0.00

	Female	3,376	0	0.00
	Total	8,285	0	0.00
Peixe-Boi (PA)	Male	4,202	0	0.00
	Female	4,083	0	0.00
	Total	12,832	0	0.00
Piçarra (PA)	Male	6,734	0	0.00
	Female	6,098	0	0.00
	Total	18,668	1	0.54
Placas (PA)	Male	9,855	1	1.01
	Female	8,813	0	0.00
	Total	24,984	22	8.81
Ponta de Pedras (PA)	Male	12,842	10	7.79
	Female	12,142	12	9.88
	Total	62,503	12	1.92
Portel (PA)	Male	32,627	9	2.76

	Female	29,876	3	1.00
	Total	40,597	2	0.49
Porto de Moz (PA)	Male	21,118	1	0.47
	Female	19,479	1	0.51
	Total	35,577	3	0.84
Prainha (PA)	Male	18,467	3	1.62
	Female	17,110	0	0.00
	Total	10,851	0	0.00
Primavera (PA)	Male	5,482	0	0.00
	Female	5,369	0	0.00
	Total	11,524	1	0.87
Quatipuru (PA)	Male	5,848	1	1.71
	Female	5,676	0	0.00
	Total	85,597	0	0.00
Redenção (PA)	Male	42,226	0	0.00

	Female	43,371	0	0.00
	Total	18,384	0	0.00
Rio Maria (PA)	Male	9,321	0	0.00
	Female	9,063	0	0.00
	Total	53,143	0	0.00
Rondon do Pará (PA)	Male	27,356	0	0.00
	Female	25,787	0	0.00
	Total	35,769	0	0.00
Rurópolis (PA)	Male	18,371	0	0.00
	Female	17,398	0	0.00
	Total	44,772	2	0.45
Salinópolis (PA)	Male	22,583	1	0.44
	Female	22,189	1	0.45
Salvaterra (PA)	Total	24,129	3	1.24
	Male	12,051	3	2.49

	Female	12,078	0	0.00
	Total	21,087	4	1.90
Santa Bárbara do Pará (PA)	Male	10,485	2	1.91
	Female	10,602	2	1.89
	Total	7,445	0	0.00
Santa Cruz do Arari (PA)	Male	3,888	0	0.00
	Female	3,557	0	0.00
	Total	73,019	2	0.27
Santa Izabel do Pará (PA)	Male	39,642	1	0.25
	Female	33,377	1	0.30
	Total	20,370	1	0.49
Santa Luzia do Pará (PA)	Male	10,240	0	0.00
	Female	10,130	1	0.99
Santa Maria das Barreiras (PA)	Total	16,548	0	0.00
	Male	8,770	0	0.00

	Female	7,778	0	0.00
	Total	24,624	0	0.00
Santa Maria do Pará (PA)	Male	12,147	0	0.00
	Female	12,477	0	0.00
	Total	32,413	0	0.00
Santana do Araguaia (PA)	Male	16,673	0	0.00
	Female	15,740	0	0.00
	Total	331,942	29	0.87
Santarém (PA)	Male	163,080	16	0.98
	Female	168,862	13	0.77
	Total	6,116	1	1.64
Santarém Novo (PA)	Male	3,205	0	0.00
	Female	2,911	1	3.44
Santo Antônio do Tauá (PA)	Total	27,461	1	0.36
	Male	13,760	0	0.00

	Female	13,701	1	0.73
	Total	16,666	2	1.20
São Caetano de Odivelas (PA)	Male	8,473	1	1.18
	Female	8,193	1	1.22
	Total	21,092	0	0.00
São Domingos do Araguaia (PA)	Male	10,716	0	0.00
	Female	10,376	0	0.00
	Total	30,599	48	15.69
São Domingos do Capim (PA)	Male	16,015	31	19.36
	Female	14,584	17	11.66
	Total	65,418	0	0.00
São Félix do Xingu (PA)	Male	34,830	0	0.00
	Female	30,588	0	0.00
	Total	14,894	0	0.00
São Francisco do Pará (PA)	Male	7,546	0	0.00

	Female	7,348	0	0.00
	Total	24,255	0	0.00
São Geraldo do Araguaia (PA)	Male	12,242	0	0.00
	Female	12,013	0	0.00
	Total	4,430	0	0.00
São João da Ponta (PA)	Male	2,287	0	0.00
	Female	2,143	0	0.00
	Total	20,689	0	0.00
São João de Pirabas (PA)	Male	10,658	0	0.00
	Female	10,031	0	0.00
	Total	13,664	0	0.00
São João do Araguaia (PA)	Male	7,290	0	0.00
	Female	6,374	0	0.00
São Miguel do Guamá (PA)	Total	52,894	10	1.89
	Male	26,181	6	2.29

	Female	26,713	4	1.50
	Total	25,643	100	39.00
São Sebastião da Boa Vista (PA)	Male	13,329	58	43.51
	Female	12,314	42	34.11
	Total	5,847	0	0.00
Sapucaia (PA)	Male	2,968	0	0.00
	Female	2,879	0	0.00
	Total	22,576	0	0.00
Senador José Porfírio (PA)	Male	12,195	0	0.00
	Female	10,381	0	0.00
	Total	24,204	2	0.83
Soure (PA)	Male	11,940	0	0.00
	Female	12,264	2	1.63
	Total	72,493	1	0.14
Tailândia (PA)	Male	36,570	1	0.27

	Female	35,923	0	0.00
Terra Alta (PA)	Total	10,400	0	0.00
	Male	5,297	0	0.00
	Female	5,103	0	0.00
Terra Santa (PA)	Total	18,782	0	0.00
	Male	9,519	0	0.00
	Female	9,263	0	0.00
Tomé-Açu (PA)	Total	67,585	5	0.74
	Male	34,412	4	1.16
	Female	33,173	1	0.30
Tracuateua (PA)	Total	28,595	2	0.70
	Male	14,704	1	0.68
	Female	13,891	1	0.72
Trairão (PA)	Total	15,242	0	0.00
	Male	8,020	0	0.00

	Female	7,222	0	0.00
	Total	39,550	0	0.00
Tucumã (PA)	Male	19,757	0	0.00
	Female	19,793	0	0.00
	Total	91,306	12	1.31
Tucuruí (PA)	Male	45,171	7	1.55
	Female	46,135	5	1.08
	Total	37,972	1	0.26
Ulianópolis (PA)	Male	19,000	1	0.53
	Female	18,972	0	0.00
	Total	43,558	1	0.23
Uruará (PA)	Male	22,812	1	0.44
	Female	20,746	0	0.00
Vigia (PA)	Total	50,832	1	0.20
	Male	25,695	1	0.39

	Female	25,137	0	0.00
	Total	58,692	1	0.17
Viseu (PA)	Male	30,472	1	0.33
	Female	28,220	0	0.00
	Total	15,607	1	0.64
Vitória do Xingu (PA)	Male	8,031	1	1.25
	Female	7,576	0	0.00
	Total	52,893	0	0.00
Xinguara (PA)	Male	26,590	0	0.00
	Female	26,303	0	0.00
